# Sympathetic Nerve Hyperactivity in the Spleen: Causal for Nonpathogenic-Driven Chronic Immune-Mediated Inflammatory Diseases (IMIDs)?

**DOI:** 10.3390/ijms19041188

**Published:** 2018-04-13

**Authors:** Denise L. Bellinger, Dianne Lorton

**Affiliations:** 1 Department of Pathology and Human Anatomy, School of Medicine, Loma Linda University, Loma Linda, CA 92350, USA; 2 College of Arts and Sciences, Kent State University, Kent, OH 44304, USA; ddifran7@kent.edu

**Keywords:** sympathetic nervous system, neural-immune, immune-mediated inflammatory diseases, adrenergic receptor signaling, rheumatoid arthritis, protein kinase A, G protein receptor kinase, mitogen-activated protein kinase, inflammatory reflex

## Abstract

Immune-Mediated Inflammatory Diseases (IMIDs) is a descriptive term coined for an eclectic group of diseases or conditions that share common inflammatory pathways, and for which there is no definitive etiology. IMIDs affect the elderly most severely, with many older individuals having two or more IMIDs. These diseases include, but are not limited to, type-1 diabetes, obesity, hypertension, chronic pulmonary disease, coronary heart disease, inflammatory bowel disease, and autoimmunity, such as rheumatoid arthritis (RA), Sjőgren’s syndrome, systemic lupus erythematosus, psoriasis, psoriatic arthritis, and multiple sclerosis. These diseases are ostensibly unrelated mechanistically, but increase in frequency with age and share chronic systemic inflammation, implicating major roles for the spleen. Chronic systemic and regional inflammation underlies the disease manifestations of IMIDs. Regional inflammation and immune dysfunction promotes targeted end organ tissue damage, whereas systemic inflammation increases morbidity and mortality by affecting multiple organ systems. Chronic inflammation and skewed dysregulated cell-mediated immune responses drive many of these age-related medical disorders. IMIDs are commonly autoimmune-mediated or suspected to be autoimmune diseases. Another shared feature is dysregulation of the autonomic nervous system and hypothalamic pituitary adrenal (HPA) axis. Here, we focus on dysautonomia. In many IMIDs, dysautonomia manifests as an imbalance in activity/reactivity of the sympathetic and parasympathetic divisions of the autonomic nervous system (ANS). These major autonomic pathways are essential for allostasis of the immune system, and regulating inflammatory processes and innate and adaptive immunity. Pathology in ANS is a hallmark and causal feature of all IMIDs. Chronic systemic inflammation comorbid with stress pathway dysregulation implicate neural-immune cross-talk in the etiology and pathophysiology of IMIDs. Using a rodent model of inflammatory arthritis as an IMID model, we report disease-specific maladaptive changes in β_2_-adrenergic receptor (AR) signaling from protein kinase A (PKA) to mitogen activated protein kinase (MAPK) pathways in the spleen. Beta_2_-AR signal “shutdown” in the spleen and switching from PKA to G-coupled protein receptor kinase (GRK) pathways in lymph node cells drives inflammation and disease advancement. Based on these findings and the existing literature in other IMIDs, we present and discuss relevant literature that support the hypothesis that unresolvable immune stimulation from chronic inflammation leads to a maladaptive disease-inducing and perpetuating sympathetic response in an attempt to maintain allostasis. Since the role of sympathetic dysfunction in IMIDs is best studied in RA and rodent models of RA, this IMID is the primary one used to evaluate data relevant to our hypothesis. Here, we review the relevant literature and discuss sympathetic dysfunction as a significant contributor to the pathophysiology of IMIDs, and then discuss a novel target for treatment. Based on our findings in inflammatory arthritis and our understanding of common inflammatory process that are used by the immune system across all IMIDs, novel strategies to restore SNS homeostasis are expected to provide safe, cost-effective approaches to treat IMIDs, lower comorbidities, and increase longevity.

## 1. Introduction

**Figure 1:**
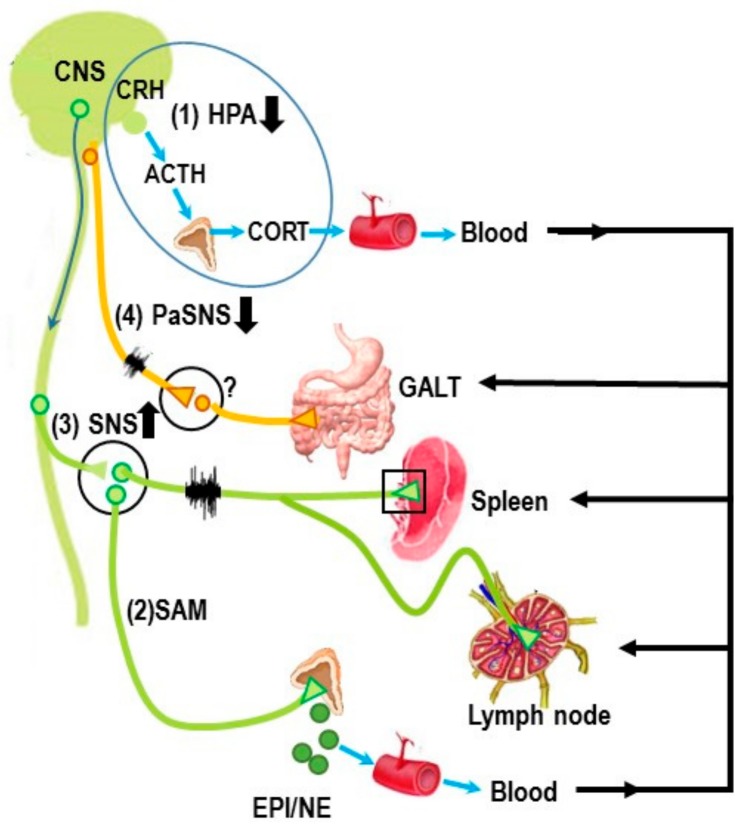
The major neuroendocrine and autonomic pathways that regulate secondary immune organs are illustrated. They include two neuroendocrine pathways, (**1**) the hypothalamic–pituitary–adrenal axis (HPA) and (**2**) the sympatho-adrenal medullary (SAM) axis, and two “hardwired” autonomic circuits, (**3**) the sympathetic nervous system (SNS) and (**4**) the parasympathetic nervous system (PaSNS). (**1**) The HPA axis is a feedback system whereby corticotropin-releasing hormone (CRH) is released from neurons in the hypothalamus that project their axons into the anterior pituitary. The release of CRH stimulates the secretion of adrenocorticotropic hormone (ACTH) into the circulation. ACTH stimulates the release of corticosterone (CORT) into the bloodstream. Circulating CORT has systemic effects including effects on secondary immune organs or tissues (Spleen, lymph nodes and gut-associated lymphoid tissue (GALT) are shown in the illustration). (**2**) In the SAM axis, preganglionic sympathetic neurons secrete acetylcholine in the adrenal medulla, which stimulates the release of catecholamines, predominantly epinephrine (EPI) and, to a much lesser extent, norepinephrine (NE), into the circulation, such as the HPA axis. Circulating NE/EPI can potentiate the actions of sympathetic nerves acting at adrenergic receptors in immune organs. (**3**) The SNS circuit to the spleen and LNs is a two-neuron chain (green neurons), with preganglionic cholinergic sympathetic neurons innervating postganglionic neurons whose nerve terminals end in the spleen and lymph nodes. The major neurotransmitter of postganglionic neurons is norepinephrine (NE). In secondary lymphoid organs, such as LNs and spleen, NE binds to adrenergic receptors expressed on immune cells, vasculature and connective tissue cells. Ligand binding to adrenergic receptors regulates the cellular responses of the immune cells that express these receptors. The predominant adrenergic receptor subtype expressed on spleen cells is the β_2_-adrenergic receptor. (**4**) The PaSNS is also a two-neuron chain (yellow neurons) of efferent nerves that supply the viscera, in particular the gut. Preganglionic neurons reside in the brainstem (medulla) and exit the central nervous system (CNS) as cranial nerves that end upon postganglionic neurons, most often embedded in the visceral organs they supply. Depicted here is the parasympathetic supply to gut-associated lymphoid tissue (GALT). There is no definitive evidence that the PaSNS innervates the spleen, but the vagus nerve clearly can influence immune responses in the spleen—most likely via the migration of vagally influenced immune cells in the gut that migrate to the spleen. The up or down arrows shown next to these regulatory pathways indicate the most common direction of change in the activity of that pathway in most of the prevalent immune-mediated inflammatory diseases, including rheumatoid arthritis.

Immune-mediated inflammatory disease (IMID) is a concept used to describe a group of highly prevalent, disabling chronic inflammatory conditions that cause end-organ tissue damage and that share the activation of common inflammatory pathways and the dysregulation of the normal adaptive immune response [1]. Table 1 lists many, but not all IMIDs. The most commonly occurring ones (in bolded text) are rheumatoid arthritis (RA), irritable bowel diseases (IBD, e.g., Crohn’s disease and irritable bowel syndrome), psoriasis and psoriatic arthritis, and systemic lupus erythematosus (SLE). These diseases share many common features that we will mention throughout the paper (summarized in Table 1), including the activation of certain inflammatory pathways that increases morbidity, co-morbidity, and premature mortality. Many IMIDs are known to be, or are suspected to be, autoimmune disorders. All IMIDs share as part of their pathophysiology an imbalance or dysregulation of the stress pathways, which are major mediators of inflammation and tissue damage (e.g., joint damage in RA). Here, we focus on the autonomic circuits (illustrated in Figure 1) and their role in chronic inflammation in IMIDs, but we acknowledge that the hypothalamic–pituitary–adrenal (HPA) axis is also involved IMID pathology (see Figure 1(1)).

The autonomic nervous system (ANS) is a major allostatic regulator essential for the normal immune responses in the spleen and other primary and secondary lymphoid organs [2,3]. Stability of the immune system is achieved through complex adaptive physiological responses that assure the demands exerted by external and internal stimuli are sufficiently met to maintain normal immune function. The immune system must adapt to acute and chronic stressors to maintain immune functions within normal ranges to sustain health and wellness. This adaptive response is necessary for survival, but can exert maladaptive “wear and tear” on visceral organ systems, and, if chronic, can affect overall health and well-being over time, a concept referred to as allostatic load [4]. The ANS directs the immune response via hardwiring of sympathetic nerves to immune cells via both α- and β_2_-adrenergic receptors and neuroendocrine regulation via the adrenal medulla. In contrast, the vagus nerves exerts an indirect action on secondary immune organs such as the spleen, as discovered recently by mobilizing Th cells from the gut that express nicotinic receptors with an alpha7 subtype (described in greater below). ANS dysregulation is a key feature of many IMIDs [5,6,7,8,9]. Aberrant ANS responses or SNS-immune cross-talk in patients with IMIDs, such as RA, or between the sympathetic nervous system (SNS) provide support for triggering, and/or exacerbating effects in many IMIDs [7,8,9,10,11,12,13,14,15,16,17,18,19,20,21,22,23,24].

Many IMIDs are not classified as autoimmune diseases, but, for many of these IMIDs, autoimmune disease are suspected or implicated in their disease processes. Moreover, chronic inflammation is a risk factor for autoimmunity due to exposure of self-antigens from tissue damage. Tissue antigens presented and processed in an inflammatory environment can break tolerance to induce autoimmunity. IMIDs, including RA, are not-pathogen driven. Persistent inflammation and hyper-immune reactivity occurs without infectious viruses or bacteria (sterile inflammation) [25]. However, viral and bacterial components are clearly associated with IA and IMIDs. These viral and bacterial components act as danger- or pathogen-associated molecular patterns (DAMPs or PAMPs, respectively), which induce inflammation, and linked with a greater risk for autoimmunity in IMIDs [25,26,27,28,29,30,31]. Our data indicate these components contribute to the dysautonomia observed in IA [32,33]. Additionally, recent findings indicate that activated sympathetic nerves release DAMPs, such as ATP, from their nerve terminals along with their vesicular neurotransmitter release [34,35,36,37,38].

**Figure 2:**
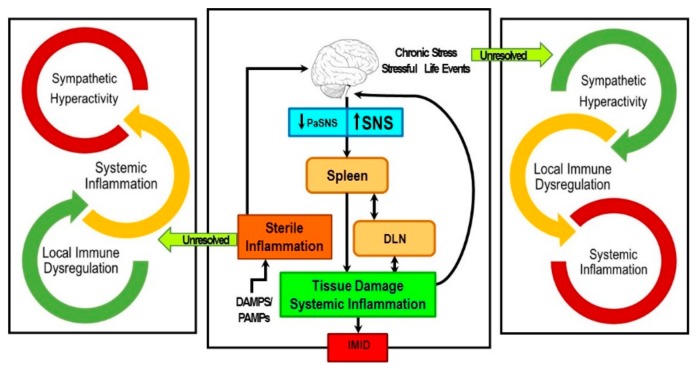
Chronic stress and/or stressful adverse life events chronically elevate sympathetic nervous system (SNS) activity and reactivity that can induce low grade local and systemic inflammation. Conversely, activation of the immune system by sterile inflammagens (danger-associated molecular patterns (DAMPs) and/or pathogen-associated molecular patterns (PAMPs)) further activates the SNS. Unresolved local and/or systemic inflammation increases SNS activity. Hyper-SNS activity (hyper-SNA) can influence/skew immune function toward a functional phenotype that can trigger or worsen immune-mediated inflammatory diseases (IMIDs), which promote visceral organ and sympathetic nerve damage. Typically, the parasympathetic nervous system (PaSNS) is suppressed. Hyper-SNA in the spleen and unresolved sterile inflammation co-conspire to precipitate and/or perpetuate an IMID. Lymph nodes drain the site of tissue damage (DLN) and influence the spleen via a lymph that drains into the spleen, and activated immune cells in the spleen migrate to the DLNs.

The spleen plays a critical role in orchestrating local and systemic immune responses that defends against foreign substances, while still protecting organ systems from “bystander damage” due to immune activation [2,3,39,40,41]. In IMIDs, unresolved sterile inflammation chronically activates the SNS and suppresses PaSNS activity [5,6]. SNS dysregulation in the presence of unresolved immune activation has been proposed as a key triggering factor for onset and disease worsening in IMIDs (Figure 2). The consequences of these chronic conditions induce cyclic and cascading hyper-sympathetic nerve activity (SNA) activity, systemic inflammation and localized immune activation in tissues targeted by the IMID. Of relevance to this Special Issue in International Journal of Molecular Science, the neural regulation of splenic immune function serves as the crossroads between localized immune responses in RA, and other IMIDs, and the systemic immune response. Spleen function is regulated by all major stress pathways, and serves as a crossroad in the pathophysiology of RA and other IMIDs. The orchestration of the immune response in the spleen determines in a major way, both the systemic inflammatory response and the localized immune dysfunction in the affected targets in RA and other IMIDs.

Since the SNS regulates systemic inflammation and immunity largely via its regulation of splenic immune functions, and maintains visceral organ allostasis, our findings raise the question: “How does hyper-sympathetic nerve activity in the spleen conspire with the immune system to induce or drive an IMID, inflammatory arthritis? There is sound biological evidence to support that maladaptive cross-regulation between these systems can drive this IMID. We present the hypothesis along with supporting research that unresolvable immune stimulation by unclearable autoantigens and DAMP or PAMP leads to a maladaptive sympathetic response to maintain allostasis, but with a health cost. The vicious “self”-targeted, continuous immune response cycle ultimately leads to chronically high SNA, and sympathetic nerve pathology in the spleen, relevant lymph nodes, and disease-targeting tissues. This paper reviews the role of sympathetic dysregulation as an important mediator in chronic IMIDs, and in particular, one of the most common IMIDs, RA. We focus on RA because the effects of the SNS on chronic inflammation and cell-mediated immune responses have been most extensively studied in this IMID, and it is an IMID of high prevalence. The purpose of this review is to provide: (1) a brief review of sympathetic dysregulation in IMIDs; (2) relevant background information on sympathetic-immune communication in the spleen as relevant to IA and IMIDs; and (3) novel findings from our research demonstrating remarkable sympathetic nerve plasticity/remodeling of the spleen and intracellular changes in β_2_-AR signal transduction in the spleen and disease-relevant lymph node cells in a well-established rodent model of RA. We critically interpret our findings and their consequences for Th cell development, Th cell function, systemic inflammation, and their clinical significance. 

## 2. Stress and ANS Dysregulation in IMIDs

The SNS and the HPA axis are the major mediators of the stress response, whereas parasympathetic activation dampens the stress response. These systems work together to coordinate basic biological activities of organ systems and to direct or redirect energy resources to organ systems to meet changing energy demands [3,4,5]. Dysautonomia is a hallmark of many IMIDs, often associated with low parasympathetic and high sympathetic nerve activity (SNA) [6,7,11,18,22,23,24,42,43,44,45,46,47,48,49,50,51,52,53,54], although other profiles have been reported, such as high parasympathetic and low sympathetic activity in asthma [55,56,57] and high parasympathetic and sympathetic activity in the murine chronic colitis model [58].

IMIDs best studied for dysautonomia are the autoimmune disorders, particularly RA. In a prospective study of RA, autonomic dysfunction is reported to precede disease development [59], and other research groups have reported that severe life stress often precedes the onset of many age-related chronic IMIDs. In RA, 80% of patients report a severe life stressor before disease onset, suggesting that severe stress can trigger the disease [24,42,43,44]. Research in an inflammatory arthritis model and other works in patients with RA indicate that a causal relationship exists between dysautonomia and IMID development and progression. In hemiplegic patients with RA, arthritis does not develop on the hemiplegic side [60,61], clearly indicating nervous system involvement. Similar phenomena are reported in other rheumatic diseases [18,61,62,63,64]. Similarly, in IA, the nervous system is required for arthritic spread to the contralateral side after injection into one foot pad of disease-inducing complete Freund’s adjuvant [65,66,67]. We found that capsaicin-induced neurotoxic destruction of small nonmyelinated afferent fibers in LNs that drain the site of an arthritis-inducing challenge in male rats prevented the transfer of disease to the contralateral side [66]. These finding support a critical role for small, nonmyelinated sensory nerves in modulating the progression of adjuvant-induced arthritis to opposing limbs.

## 3. Autonomic Innervation of the Spleen and Systemic Inflammation

Research overwhelmingly indicates bidirectional interactions between the ANS and immune system to regulate peripheral systemic inflammation [2,45,52,68,69]. Main targets of the SNS where systemic inflammation is regulated are cardiovascular system, gastrointestinal system, and the spleen, of which the latter is interposed within the cardiovascular system as a blood filtration device. Anatomical connections via nerves to blood vessels and immune organs, as well as neuroendocrine system, provides the substrate that links psychosocial stress with changes in immune function and sickness behavior after immune activation. The PaSNS also influences splenic immune function, but the majority of data support it does so indirectly. The ANS also influences local inflammatory responses in visceral and somatic tissues, via adrenergic and cholinergic receptor-mediated regulation of immune cells, connective tissue, and cardiovascular functioning. The ANS plays a major role in fat cell metabolism, vascular remodeling, and fibrosis associated with chronic inflammation [10,70,71,72,73,74,75]. The prevalence of chronic inflammation increases in frequency with advancing age, and may significantly contribute to premature aging, for which the term inflammaging has been coined [76,77].

The spleen is ideally positioned within the circulatory system as a blood filter in vertebrates and serves to detect, respond to, and protect against blood-borne antigens, damaging cytokine mediators and inflammagens from immune activation [78,79,80,81,82,83]. The interface site between the non-lymphoid red pulp and the lymphoid white pulp of the spleen is the marginal zone, a transitional site from circulation to peripheral lymphoid tissues. A key role of the marginal zone is to trap particulates and initiate innate responses against them, or to initiate immune tolerance. It is an important site for driving a coordinated response between multiple myeloid phagocyte and lymphocyte subsets. Sympathetic nerves present at this site coordinate these immune activities. For example, indirect activation of vagal afferent fibers to increase sympathetic nerve activity attenuate macrophage TNF-α production from systemic challenge with LPS via a circuit that involves the vagus nerve [41].

Through its systemic anti-inflammatory cytokines responses, the spleen can protect the organism against potentially damaging and life-threatening pro-inflammatory “cytokine storms” that ensues from a systemic infection or from the unresolved immune responses in targeted tissues in IMIDs. In contrast, local tissue fluid drains into lymph nodes via lymphatic vessels; lymph nodes survey and detect foreign antigens in the local tissue fluids [84,85]. Lymphocytes recirculate from the blood, through lymph nodes, into lymphatics and back to the blood. Lymph and immune cells that drain via lymphatic vessels to regional lymph nodes (DLNs) eventually enter the bloodstream, which provides an important route of coordinating communications between regional immune responses in lymph nodes and systemic immunity regulated by the spleen. Both secondary immune organs play significant roles in the development and progression of IMIDs, and, as discussed in the later sections, are differentially regulated by sympathetic nerves in inflammatory arthritis and other IMIDs.

### Autonomic Circuitry to the Spleen: Anti-Inflammatory Pathways

Central-mediated stress responses influence splenic immune functions by direct sympathetic innervation of lymphoid tissue and via hormones that enter the bloodstream [86,87,88,89]. Sympathetic nerves distribute to immune compartments in the spleen, and manipulation of the splenic nerve or its activity alters splenic immune responses, as well as distant localized and systemic inflammation. Splenocytes express adrenergic receptors (predominantly β_2_) and receptors for other neural and endocrine mediators, and so they are able to detect and respond to these signals as they are released from nerves or pass in the blood circulating through the spleen [90]. These ligand–receptor interactions regulate the development, movement, expansion, and function of every type of immune cell in the spleen. Thus, the SNS regulates cytokines/chemokines/trophic factors secreted by immune cells that enter the bloodstream to act both peripherally and centrally. The outcome of this neural-immune communication aims to integrate environmental and endogenous challenges to permit immune adaptations to inflammatory events in real time. Maladaptive plasticity, for example hyper-SNS activity/reactivity or excessive neuroendocrine mediators, may be at the core of stress-promoting inflammaging and the development of chronic inflammatory conditions.

The sympathetic anti-inflammatory pathway comprises a complex integrated efferent circuit between the SNS and immune system [2]. Autonomic innervation of the spleen is supplied solely by sympathetic nerves that derive from the superior mesenteric-celiac ganglion (SMCG) [91]. These nerves supply the white pulp, where cells of the immune system reside, and blood vessels, including the venous sinuses. These nerves avoid the parenchyma of the red pulp and B cell follicles in the rodent spleen. In the periarteriolar lymphatic sheath, sympathetic nerves course among T cells, but in the marginal and parafollicular zones they are closely apposed to macrophages, B cells and T cells.

Functionally, systemic LPS injection activates sympathetic nerves to the spleen [92], and suppresses systemic inflammation, and thus has been referred to as the anti-inflammatory pathway. This anti-inflammatory response is most commonly assessed by macrophage production of TNF-α levels in spleen, serum and liver. The spleen is required for this response [39,40], and when the spleen is removed, the plasma levels of inflammatory cytokines are reduced in its absence. Thus, the spleen is the main source of pro-inflammatory cytokines induced by intravenously administered LPS, and is the site of vagal-mediated pro-inflammatory cytokine suppression. Moreover, the destruction of sympathetic nerves in the spleen or norepinephrine depletion by treatment with reserpine prevents the vagally-stimulated anti-inflammatory response [36], demonstrating the necessity of noradrenergic sympathetic neurotransmission of this response.

Although the anti-inflammatory pathway has been referred to as cholinergic [93,94], it is technically a misnomer, because neither the afferent sensory nor efferent postganglionic sympathetic neurons that supply the spleen release acetylcholine [69,95]. Efferent preganglionic sympathetic neurons that innervate postganglionic neurons are all cholinergic and their release of ACh binds to nicotinic receptors expressed on postganglionic neurons. Either central muscarinic agonists (icv.) or peripheral nicotinic administration can stimulate cholinergic efferent vagal nerves that indirectly drive the anti-inflammatory activity of sympathetic nerves in the spleen to suppress local and systemic inflammation [69,95,96,97,98,99]. Interestingly, atropine, a peripherally-acting muscarinic blocker, does not block the anti-inflammatory effect of the centrally applied muscarinic agonist. Taken together, these findings indicate that the vagus nerve is an important substrate for the central action of muscarinic agonists in the suppression of inflammation. Additionally, electrically stimulating the distal end of the severed cervical vagus nerve that inhibits local and systemic inflammation induced by LPS does not drive the firing of the splenic nerve [100]. The anti-inflammatory response to i.p. challenge with LPS also depends on the integrity of sympathetic nerves in the spleen [41,101], the main source of pro- and anti-inflammatory cytokines induced by a systemic challenge, like LPS.

The anti-inflammatory response by vagal stimulation is mediated via α7ChR (α7 nicotinic cholinergic receptor), because mice lacking the α7 nicotinic receptor (α7ChR) subunit fail to generate an anti-inflammatory response to intraperitoneal challenge with LPS [101,102]. Nicotinic antagonists or β-AR agonists suppressed in vitro TNF-α production by macrophages from wildtype but not from the α7 knockout mice, inferring macrophages as the source of TNF-α [102]. However, vagally-mediated suppression of LPS-induced inflammation requires T lymphocytes, as vagal stimulation fails to suppress inflammation in nude mice, but can be partially restored by the adoptive transfer of T lymphocytes that produce ACh into nude mice [103].

Collectively, in the rodent spleen there is little credible evidence for direct parasympathetic innervation of the spleen. The majority of studies that use anatomical tracing, neurochemical, and physiological techniques support that the spleen receives NO significant vagal afferent or efferent nerve supply [100,104,105]. However, the functional data indicate that the vagus nerve can regulate the sympathetic activity in the spleen to indirectly promote the anti-inflammatory actions of the efferent sympathetic anti-inflammatory response by a unique route that is, in part, a non-neural link conveyed by α7AChR-positive memory T lymphocytes based on high CD44 and low CD62L expression. The data support that vagal stimulation induces the migration of T cells from as yet an unidentified site, where their secretion of ACh in the spleen suppresses sympathetically-mediated suppression of TNF-α secretion, possibly by ACh binding to presynaptic α7ChR expressed on sympathetic nerve terminals. This mechanism of action has been demonstrated for vagal nicotinic-induced nitric oxide-mediated vasodilation of the vasculature, but requires testing in the spleen [106]. Another indirect vagal-sympathetic pathway has been described [107], whereby vagal sensory afferents are directly activated by catecholamines (epinephrine and norepinephrine), providing a centrally-mediated negative feedback signal to adjust the activity of the sympathoadrenal system. Circulating epinephrine (and to a lesser extent norepinephrine) secreted from the adrenal glands reinforces the neural activity that stimulates adrenergic receptors expressed on cells of the immune system and vasculature in the spleen. This arm of the SNS is largely responsible the widespread systemic stress response mediated via neuroendocrine catecholamines (NE and EPI) release from the adrenal medulla into the circulating blood.

Further discussion is warranted regarding the sympathetic anti-inflammatory pathway, as they relate to Tracey’s coining of the term, “inflammatory reflex”, to describe a CNS response to reflexively suppress systemic inflammation due to a lethal or high sublethal dose of LPS as a model of sepsis. All reflexes have an afferent limb in addition to the central processing and efferent components, but in all of Tracey’s models of the inflammatory reflex, the neural hardwired afferent limb is conspicuously missing. In fact, very little is understood about the afferent limb of the inflammatory reflex. Is the afferent limb hardwired or humorally mediated? Could it be dose- and/or route-dependent? Is the afferent limb the same as that described for the LPS-induced humoral pathway that activates the HPA axis to induce fever and other sickness behaviors [68,108]? If so, then activation of the HPA axis should be investigated as a mediator of the “anti-inflammatory reflex”, as well. Borovikova and coworkers [96] initially reported that bilateral vagotomy increased plasma TNF-α 40% in rats treated intravenously with LPS (15 mg/kg), but they also found a 33% reduction in corticosterone in these rats. Therefore, the possibility that reduced circulating glucocorticoid levels significantly contributed to the pro-inflammatory response cannot be discounted [68,109]. The dose of LPS given does seem to be an equally important variable in eliciting an anti-inflammatory response, as lower doses of intravenous LPS (8 mg/kg) significantly reduced plasma TNF-α levels by ~50% [110]. This unresolved question as to whether vagal-mediated anti-inflammatory responses constitute an efferent pathway of a reflex that is triggered by an inflammatory stimulus raises serious concerns for advancing vagal electrical stimulation to the clinical setting.

## 4. Sympathetic Innervation of the Splenic T Cells Is an Important Mediator of Chronic Inflammation in the IMID, Inflammatory Arthritis

Progression of adjuvant-induced in male Lewis rats drives sympathetic nerve plasticity and remodeling in the white and red pulps in the spleen. Sympathetic nerves retract during acute disease, but later return to the white pulp and also sprout into the adjacent red pulp, a compartment of the spleen where these nerves are sparse [111,112,113,114]. This finding combined with cellular mobilization, migration proliferation and activation suggests novel nerve-immune cell targeting patterns that likely have functional significance. Similarly, sympathetic neuropathy is reported in the arthritic joints of rodents with inflammatory arthritis [115] and in the pancreas of rodent models with autoimmune diabetes and islet-selective loss of sympathetic nerves in the pancreas of humans with Type-1 diabetes [116,117]. The p75 neurotrophin receptor which is present on sympathetic axons plays a mediating role in islet sympathetic neuropathy [118].

Consistent with findings in RA patients [60], sympathectomy lessens disease severity in rats with adjuvant-induced arthritis [118]. In contrast, rat with spontaneous hypertension that result from sympathetic nerve hyperactivity develop more severe arthritis [119]. There seems to be two phases of SNS reactivity, such that, early in disease progression, the SNS promotes inflammation, whereas, in late disease phase, the SNS exerts anti-inflammatory actions [120,121,122,123]. During both phases of disease, the effects of the SNS are mediated by both α- and β-ARs [32,120,124]. During early adjuvant-induced arthritis, the SNS promotes greater Th1- and Th17-type of immune responses [125].

Pro-inflammatory actions of the SNS in the early phase of collagen-induced arthritis are mediated by CD25+FOXP3- Th cells [123]. These findings in rodent models appear to contradict findings from human studies, but are likely explained by the timing of changes in SNS reactivity. In general, blocking sympathetic activity early after challenge, either by sympathectomy or therapeutics, attenuates disease, but blocking sympathetic activity during severe chronic disease worsens or accelerates disease activity [120]. The reason for such dichotomous outcomes are not well established, but could in part result from an interplay between changes in sympathetic nerve density and function in the spleen across disease course, and the depletion of sympathetic (and/or sensory) nerves in the inflamed synovium as disease progresses [122,126,127]. This hypothesis is consistent with findings in RA patients that hemiplegia prior to RA onset can prevent arthritis from developing in joints on the paralytic side of the body [61,64,128].

In RA, a prototypic IMID, there is autonomic imbalance/dysfunction that precedes and predicts arthritis development in subjects at risk of developing seropositive RA [51,129,130]. RA patients that have relatively high vagal nerve activity (based on heart rate variability) have better outcomes with anti-rheumatic treatments. Electrically stimulating the vagus nerve or treatment with an α7nACR agonist reduces the signs and symptoms of inflammatory arthritis in a rodent model, and the production of pro-inflammatory cytokines that erode the affected joints [51]. These findings are consistent with a role for the vagus nerve in modulating sympathetic activity in the spleen to negatively affect disease, similar to what has been described in murine sepsis models. The implications of this work are the potential use of bioelectrical approaches to treat chronic inflammatory disease such as RA and other IMIDs.

Acute inflammation typically induced by i.p. injection of LPS: (1) increases sympathetic nerve activity (SNA) in the spleen [92,131]; (2) induces an anti-inflammatory response by suppressing circulating TNF-α and/or IL-1β, and increasing anti-inflammatory cytokines like IL-10; (3) inhibits Th1 cell differentiation by suppressing IFN-γ production; and (4) limits T-helper cell expansion by reducing the IL-2 production. This response profile is largely mediated by β_2_-AR activation in immune cell targets of sympathetic nerves. The source of TNF-α and IL-1β is antigen-presenting cells, and IL-2 and IFN-γ are from T cells; the source of secreted IL-10 is not fully resolved.

The β_2_-AR-mediated secretion of this cytokine response pattern is mediated via Gs protein-coupling to adenylate cyclase, which increases cAMP and the protein kinase A (PKA) activity. Our research in a rodent autoimmune model of RA supports that under chronic inflammation and autoimmunity, β_2_-AR signal transduction is altered in spleen cells and in disease-relevant lymph nodes [89,124], such that the β_2_-AR uses GRKs and β-arrestin pathways instead of cAMP-PKA to mediate spleen cells responses during the effector phase of inflammatory arthritis. The specific details of this signaling switch are presented in detail below. The consequences of signal switching on disease outcome remain to be fully determined, but it is clear that the switch to GRK mediation significantly alters cytokine profiles differentially in spleen and DLNs, and therefore is likely contributory to the early vs. late effects of sympathetic nerve manipulation discussed earlier in this paper. Moreover, if β_2_-adrenergic signal switching is demonstrated in RA and other IMIDs it may add another level of complexity that makes it more difficult to predict the outcome of vagus nerve stimulation on sympathetic regulation of pro-inflammatory cytokine secretion in the spleen.

## 5. IMIDs Induce SNS Pathology in Lymphoid Organs

IMIDs induce sympathetic nerve pathology in the secondary lymphoid organs including the spleen, the lymph nodes that drain targeted sites of inflammation, and disease-targeted sites of chronic inflammation. For example, we and others have reported sympathetic neuropathy in the spleen and affected joints in murine models of lupus [90,132,133,134]. In humans, high SNA and/or sympathetic neuropathy are evident in diabetes [47], hypertension [47], heart failure, and COPD [135,136]. In an animal model of RA, we have observed dying back of sympathetic nerves in immune compartments of the spleen coupled with reduced naïve ED3+ macrophages and CD4+ Th cells in the white pulp and loss of IgM+ B cells in the B cell follicles [111,112]. A greater abundance of sympathetic nerves was apparent in the red pulp in areas of NGF-positive macrophages [111,112]. We propose that the dying back of sympathetic nerves from the white pulp and subsequent sprouting of these nerves into the red pulp is driven by neurotrophins secreted in the reorganized red pulp as the disease progresses. Remodeling of sympathetic nerves suggests novel targeting of immune cell populations residing in the red pulp. These findings are indicative of an injury and sprouting response of sympathetic nerves that reduces innervation/regulation of normal naïve target cells in the white pulp and abnormal innervation of more activated immune cells (macrophages, and perhaps antibody producing B cells) in the red pulp. Such changes would be expected to promote disease.

There is also evidence for altered SNS regulation of macrophage and Th cell functions in IMIDs, mostly a loss in the ability of the SNS to inhibit inflammatory and cell-mediated immune responses. In some cases, the SNS is reported to promote inflammation and cell-mediated immunity rather than to restore immune system homeostasis, a finding easily explained by classical β_2_-AR signaling pathways. These findings are consistent with reports of altered β_2_-AR receptor signaling in macrophages and Th cells in IMIDs. 

## 6. β_2_-Adrenergic Receptors: “Friend or Foe” in IMIDs?

### 6.1. Canonical Signaling Pathway: “Friend”

**Figure 3:**
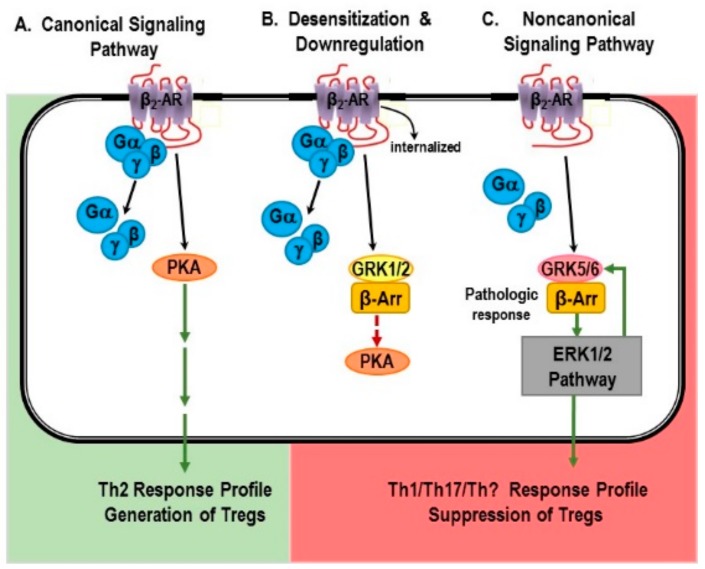
Normal β_2_-adrenergic receptor (β_2_-AR) signal transduction via G-coupled proteins (canonical), β_2_-AR desensitization and downregulation, and alternative signal transduction mediated by β-arrestin (β-Arr) are shown in (**A**–**C**), respectively. (**A**) The Canonical pathway: Ligand binding to the β_2_-AR causes dissociation of G-protein to Gα and Gβγ and activation of protein kinase A (PKA). Traditionally, activation of this signaling pathway promotes a Th2-type immune response and an increase in Treg cell function that promotes immune response resolution and suppression of inflammation. (**B**) Desensitization and Downregulation: Chronically high SNA in the spleen desensitizes and down-regulates β_2_-AR via G protein-coupled receptor kinase (GRK)1/2/β-arrestin (β-Arr) signal transduction that inhibits the p38MAPK pathway, ERK1/2, which prevents PKA activity. (**C**) The activation of the non-canonical β_2_-AR signaling pathway is mediated by a non-G protein mechanism (G protein is uncoupled to the receptor). Instead, GRK5/β-arrestin (β-Arr) signal transduction activates the ERK1/2 signaling pathways. The consequences for these two signaling pathways for immune responses in the spleen are indicated below. Signal transduction by the PKA pathway promotes a Th2-type immune response and the generation of Treg cells. In contrast, β_2_-AR signaling via the ERK1/2 pathway drives Th1 and/or Th17 immune responses depending on the immune stimulus, and suppression of Treg cell function.

In the canonical pathway, NE binding to β_2_-ARs induces an increase in intracellular cAMP and ultimately PKA (Figure 3A) [137,138,139,140,141]. These receptors are G-protein coupled receptors (GPCRs) that are associated specifically to Gαs. NE binding to β_2_-ARs expressed on immune cells causes the exchange of receptor associated guanosine diphosphate (GDP) for guanosine triphosphate (GTP), which activates cAMP-PKA-mediated intracellular signaling [137,138,139,140,141]. Briefly, NE binds to β_2_-ARs expressed in immune cells. Ligand binding induces guanosine diphosphate–guanosine triphosphate (GDP–GTP). This event triggers the uncoupling of the Gαs and Gβγ components, and the recruitment of GTP-Gαs to the membrane. GTP-Gαs then activates adenylate cyclase (AC). AC catalyzes the conversion of ATP to cAMP, the second messenger for β_2_-ARs [142,143]. Cyclic-AMP activates PKA, a kinase that mediates most of the resulting gene transcription that occurs with β_2_-AR activation. Additionally, cAMP can activate gated ion channels, and the exchange protein, Epac (exchange protein directly activated by cAMP; not shown in Figure 3) [144]. Signaling ends when cAMP is degraded by phosphodiesterases (PDE). Stimulation of β_2_-ARs is well-known to suppress myeloid- and T lymphocyte-mediated inflammation via the canonical pathway.

Signaling via cAMP-PKA is terminated by phosphorylation of β_2_-ARs by PKA, which induces a conformational change in the receptor that impairs receptor coupling to Gs. Thus, uncoupling of the receptor from Gs results in receptor desensitization (Figure 3B). Phosphorylation by GRK1/2, which recruits β-arrestin-1 to the receptor, induces receptor internalization. Once internalized, β_2_-ARs are either recycled to the membrane or trafficked to lysosomes for degradation, the latter being referred to as receptor down-regulation. Receptor desensitization and down-regulation are observed under high NE concentrations and high sympathetic nerve activity.

The regulation of β_2_-ARs by GRKs and β-arrestins plays essential roles in maintaining the normal functions of cells and tissues, including the immune cells. Cells of the immune system express high levels of GRK2, exceeding levels in the heart by 4–5-fold [145]. While essential for normal SNS functions, under chronic high SNS activity driven by a robust long lasting inflammatory or chronic cellular immune response, receptor desensitization and down-regulation can remove an important negative feedback loop required for terminating the immune response and restoring homeostasis. Down-regulation and desensitization of the β_2_-AR is observed in many diseases where net SNS firing rate (activity) is chronically elevated and robust/chronic inflammation ensues, including hypertension [146], sepsis [147,148,149], RA [150,151,152], and asthma [153,154,155]. Down-regulation, desensitization, and shifts in receptor signaling have not been extensively examined in psoriatic arthritis, psoriasis, IBDs, RA or SLE. However, research does support increased SNA in these disorders, as previously stated, an event that is known to down-regulate, desensitize and even shift β-adrenergic receptor signaling. Altered β_2_-AR functions is reported for all of these diseases, including, lack of receptor ability to induce cAMP/PKA activation or reduced receptor density in immune cells and cells targeted by the disease [18,153,156,157,158]. Further, polymorphisms in β_2_-ARs and abnormal Gs protein, the traditional G-protein linked with these receptors, are reported in psoriasis [20,159]. Evidence also exists for altered β_2_-AR signaling in spleen lymphocytes in an animal model of ulcerative colitis [160]. In spleen lymphocytes, β_2_-AR-β-arrestin 2-ERK1/2 signal transduction pathway participated in the pathologic course of ulcerative colitis. Activation of the ERK1/2 signaling pathway cannot be easily explained by signaling via the canonical pathway for β_2_-AR. Whether these changes in receptors are a compensatory response required for survival at the expense of optimal health, or a maladaptive response that further promotes disease processes is not known.

#### 6.1.1. Non-Canonical Signaling Pathway: “Friend or Foe”? 

Over the last decade, it has become clear that stimulation of β_2_-ARs can also activate MAPK (also known as extracellular signal-regulated kinase (ERK)) pathways in non-immune system cells (e.g., cardiac myocytes and endothelial cells) and in cultured cell lines [161,162,163,164,165]. Whether these non-canonical signaling pathways can be activated by β_2_-ARs in immune cells is just beginning to be discerned. However, activation of these pathways in immune cell could explain many observations in the literature where agonist-stimulated β_2_-ARs promote and drive inflammation and cellular immunity. It is also unclear whether shifts in signaling from the cAMP-PKA pathways to signaling pathways that promote inflammation and cellular immunity is beneficial or detrimental, but is likely to be both depending upon the circumstances (e.g., type of antigen challenge, capacity for antigen clearance, type of immune response mounted). 

#### 6.1.2. The β-AR Switch to Non-Canonical MAPK Pathways

Site-specific phosphorylation by specific subtypes of GRKs determines whether the GPCRs undergo desensitization or signal via an alternative pathway [166]. It has been repeatedly demonstrated in various cell types that, when there is high physiologic or chronic receptor stimulation, β_2_-ARs can signal via a G protein-independent, GRK5 or -6 (GRK5/6) and β-arrestin-2-dependent non-canonical signaling pathway [161,162,163,164,165]. In many cell types, shifts in β_2_-AR signaling MAPK pathways occurs with phosphorylation of the receptor by PKA and GRK5/6. These shifts in β_2_-AR signaling directly oppose the largely inhibitory effects mediated by cAMP [167,168] and are expected to promote inflammation and cellular immunity since these events are driven by ERK1/2 and MAPK pathways. While data for this occurring in immune cell are sparse, recent studies support that β_2_-AR signaling in macrophages and Th cells exists (described below). Immune cells do express GRK5 and -6 [145,169], but these GRKs are less well studied than GRK2 in these cells. However, this is a fertile area of research, as activation of these pathways in immune cell could explain many observations in the literature where agonist-stimulated β_2_-ARs promote and drive inflammation and cellular immunity. It is also unclear whether shifts in signaling from cAMP-PKA to pathways that promote inflammation and cellular immunity are beneficial or detrimental, depending upon the circumstance. 

#### 6.1.3. Mechanisms for Receptor “Switching”

**Figure 4:**
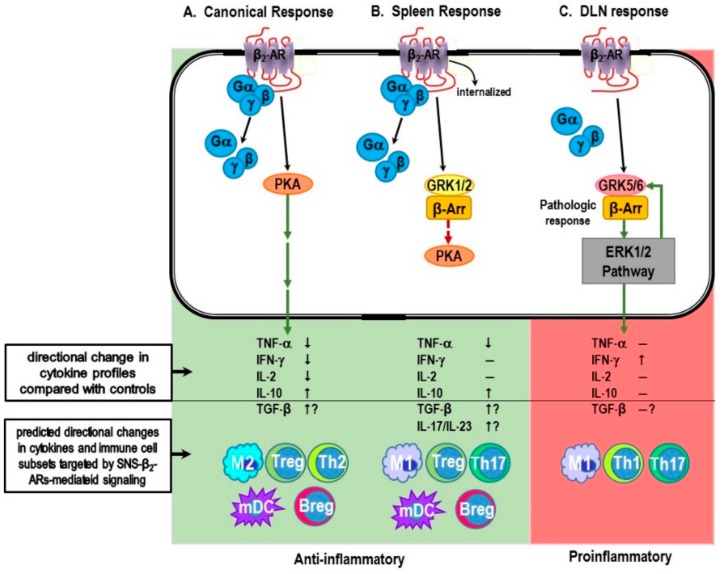
(**A**) Canonical β_2_-adrenergic receptor (β_2_-AR) signaling response, the anti-inflammatory cytokine profile mediated by protein kinase A (PKA), and the major anti-inflammatory cellular mediators of the resulting anti-inflammatory immune response. (**B**) The physiological G protein-coupled receptor kinase (GRK)1/2-mediated β_2_-AR desensitization/downregulation in spleen cells from rats with inflammatory arthritis, the reported directional changes in cytokine profiles compared with controls, and the predicted cellular mediators of the resulting immune response. (**C**) The pathological GRK5/6-mediated activation of the ERK1/2 pathway in immune cells from lymph node cells that drain the affected joints (DLN cells) from rats with inflammatory arthritis, the reported directional changes in cytokine profiles compared with controls, and the predicted cellular mediators of the resulting immune response. Predicted immune cell mediators are based on well-documented functions and cytokine profiles secreted by specific populations of immune cells, particularly the secretion of the anti-inflammatory cytokines, interleukin (IL)-10 and tumor growth factor-β (TGF-β) by regulatory B and T cells (Breg and Treg, respectively), IL-10 by myeloid dendritic cells (mDC), tumor necrosis factor (TNF)-α by macrophages, IL-2 by Th2 cells, and interferon (IFN)-γ by T-helper (Th)1 and Th17/Th1 cells. Predicted M1 and M2 macrophages are based on their ability to drive a Th1/Th17 or Th2-type immune responses. SNS, sympathetic nervous system.

Shifting β_2_-AR signaling from cAMP to ERK1/2 MAPK pathway is initiated by phosphorylation of the receptor by GRK5/6. GRK5/6 recruits β-arrestin 2 to the receptor. GRK5/6 and β-arrestin-2 induce the receptor to be internalized in the cell within endosomes. Receptors phosphorylated by GRK5/6 with subsequent β-arrestin-2 recruitment generate receptor-initiated signals that are G protein-independent and β-arrestin-2-dependent. Beta-arrestin 2 provides a scaffold for ERK1/2 activation. Briefly, under high SNA and high NE concentrations, PKA and GRK5/6 phosphorylate the carboxy-terminus of the receptor. This allows β-arrestin 2 to bind to the β_2_-AR (Figure 4C) and sets up the scaffolding for ERK1/2 activation [164,166,170,171,172,173]. ERK1/2 activation induces the activation of transcription factors, which travel to the nucleus to alter gene transcription. Thus, high agonist concentrations can induce sustained ERK activation independent of the G protein pathways [164,174,175]. Therefore, chronically elevated sympathetic tone provides conditions for differential phosphorylation of β_2_-ARs that result in sustained β_2_-AR-induced ERK1/2 signaling. Signal transduction via ERK1/2 may explain why β_2_-AR stimulation can up- or down-regulate certain responses such as lymphocyte proliferation or production of cytokines that control the T cell subsets that differentiates in an immune context-dependent manner.

In summary, the subtype of GRK that phosphorylates the receptor determines the functional role of β-arrestin [166]. Phosphorylation by GRK2 induces β-arrestin 1-mediated desensitization, whereas phosphorylation by GRK5 or -6 results in β-arrestin 2-mediated signaling. Phosphorylation of the β_2_-AR by GRK2 or GRK5/6 is dependent on the concentration of the agonist and duration of agonist exposure [165,176,177]. High agonist concentrations induce GRK-5/6-phosphorylation of the β_2_-AR and signaling towards non-canonical signaling pathways. In contrast, high agonist concentrations induce receptor desensitization and downregulation when the receptor is phosphorylated by GRK2. Whether the receptor is phosphorylated by GRK2 or GRK5/6 is context dependent. 

### 6.2. Evidence for Non-Canonical Pathway Signaling in Immune Cells

Most studies determining the role of NE or β_2_-AR agonists on cell functions involved in innate and adaptive immunity report immunosuppressive effects (reviewed in [2,105]). However, there are important contexts in which β_2_-AR stimulation of immune cells enhances inflammation and cellular immunity in vivo and in vitro. These studies support the physiologically relevance of “signal switching” in thwarting pathogens and in some diseases.

#### 6.2.1. Myeloid Cells and Innate Immunity

Szelenyi et al. [178] were the first study to show that activation of β_2_-ARs inhibit or promote inflammatory responses in monocytes and macrophages in a context dependent manner. They found that stimulating these cells with different myeloid cell activators, lipopolysaccharide (LPS) or phorbol myristyl acetate (PMA,) in combination with β-AR agonist-induced decreases or increases, respectively, in TNF-α, IL-12, and nitric oxide production compared to cells treated in the absence of the β_2_-AR agonist. The inhibiting and enhancing effects, respectively, of the β_2_-AR agonist were blocked by a β-AR antagonist, demonstrating that the effects were mediated by β_2_-ARs. In LPS stimulated cells, the β_2_-AR agonist reduced activation of both MAPKs, ERK1/2 and p38. This is consistent with findings that LPS promotes adenylyl cyclase activity [179], suppresses translocation of GRK2 to the membrane [180], and reduces the expression of GRK5 and -6 [181], effects expected to promote signaling via the cAMP-PKA pathway. Changes in GRK2, -5 and -6 are mediated via activation of ERK1/2, indicating cross talk between MAPK pathways and GRKs that regulate GPCR functions and provide mechanisms by which LPS, in addition to catecholamine-dependent regulation, can impact β_2_-AR functions.

In contrast, mechanisms by which PMA augments macrophage production of TNF, IL-12 and nitric oxide are not clear. In contrast to LPS and β_2_-AR agonist treatment, PMA and β_2_-AR agonist treatment enhanced p38, but had no effect on ERK1/2. Further, activation of p38 occurred more rapidly and robustly and with shorter duration with PMA compared to LPS treatment. The differences in MAPK activation, kinetics and magnitude between PMA and LPS result in the opposing β_2_-AR agonist induced cytokine production. The extent that this mechanism is responsible for the difference in receptor function remains unclear. Alternatively, PMA directly activates protein kinase C (PKC) which can impact receptor signaling pathways in several ways. PKC also phosphorylates β_2_-ARs at the same site as PKA, thus, promoting receptor desensitization [176,182,183], but has reduced efficacy. PMA inhibits adenylyl cyclase activity [184], which limits cAMP production. In contrast, PKC suppresses GRK2 activity and promotes GRK2 trafficking to the membrane [185,186,187], events expected to suppress receptor desensitization and down-regulation. These effects of PMA should suppress activation of c-AMP induced by β_2_-AR. However, PMA also phosphorylates GRK5, suppressing its ability to phosphorylate β_2_-ARs [188], an effect that should limit signaling through the non-canonical pathway. These effects of PMA do not adequately explain the observed increases in β_2_-AR agonist-induced production of TNF-α, IL-12 or nitric oxide in PMA-activated macrophages. Thus, β_2_-ARs suppress LPS-induced, but enhance PMA-induced inflammatory responses.

These findings support that β_2_-ARs can act as a “molecular switch” to either inhibit or promote myeloid cell driven inflammatory responses, depending upon the context for the challenge.

Another potential mechanism for β_2_-AR potentiation of macrophage cytokine production through ERK-mediated effects in PMA signaling of the NF-κB pathway. NF-κB is an inducible transcription factor that regulates gene expression of several inflammatory cytokines, including TNF-α [189]. Recent studies indicated PMA phosphorylates IκBα which removes its inhibition of NF-κB nuclear translocation. This allows NF-κB to enter the nucleus, bind to DNA, and increases pro-inflammatory cytokine production, including TNF-α [190,191]. An NF-κB binding site is present in the promoter region of GRK5 that is consistent with the observed increases in GRK5 levels in PMA treated myocytes [191]. Increases in PMA-induced GRK5 levels were abolished with the addition of an NF-κB or IκB inhibitor. These findings provide a potential mechanism to explain β_2_-AR agonist induced increases in TNF-α, IL-12 and nitric oxide production by PMA-differentiated monocytes [178]. The PMA-induced GRK5 is expected to phosphorylate β_2_-ARs, which promotes signaling via a β-arrestin 2-mediated activation of ERK1/2, a signaling pathway well known to amplify production of TNF-α. Future studies are needed to test this hypothesis.

#### 6.2.2. T Cells and Adaptive Immunity

There is evidence that, in addition to activating the cAMP-PKA pathway, β_2_-AR in CD4^+^ T cells can signal via non-canonical MAPK pathways. CD4^+^ T cells function in adaptive immune responses, promoting either a cellular or humoral immune response. Naïve CD4^+^ T (T helper (Th)0) cells differentiate into either Th1 or Th2 cells, which drive cellular or humoral immunity, respectively [192]. Beta_2_-AR-induced cAMP directly regulates the differentiation of Th0 cells to Th1 cells by inhibiting IFN-γ, a cytokine required for Th1 cell development. Lowering IFN-γ indirectly promotes development of Th2 cells by removing the inhibitory effects of this cytokine on Th0 cell IL-4 production, a cytokine required for Th2 cell differentiation. This is consistent with the expression of β_2_-AR on Th0 and Th1 cells and their absence on Th2 cells. Beta_2_-ARs also inhibit Th1 cell production of IL-2, a cytokine required for Th1 cell proliferation and expansion. Thus, treatment of antigen-activated CD4+ Th0 cells with NE or selective β_2_-AR agonists suppresses IFN-γ and IL-2 (reviewed in [192]), an effect that limits the ability of Th0 cells to produce IFN-γ, proliferate and develop into Th1 cells. These findings have led to the generalized concept that the SNS suppresses cell-mediated immunity (reviewed in [33]).

In contrast to this generalized concept, the SNS can promote cellular immunity. A concept that makes sense considering the question, “why would the SNS inhibit a cell-mediated immune response during a bacterial infection”. This seems counter-intuitive for a system that maintains homeostasis and induces stress responses to antigen challenges. Studies have indeed reported that the SNS can promote cellular immunity. Stimulation of β_2_-AR expressed in naïve CD4+ Th0 cells increases the production of IFN-γ [193], when the receptors are activated before or just after the immune challenge with an antigen or anti-CD3CD28 co-stimulation. The increased production of IFN-γ enhances Th1 cell proliferation and differentiation, promoting rather than inhibiting cellular immunity [193]. Thus, stimulation of β_2_-ARs before antigen activation reduces IFN-γ synthesis, whereas β_2_-agonist stimulation after antigen challenge increases IFN-γ production to promote cellular immunity and pathogen removal. 

In our rat model of RA, we also observed that stimulation of β_2_-ARs can enhance T cell IFN-γ production, providing evidence for a shift in β_2_-AR signaling from cAMP to MAPK pathways in lymphocytes [32,124]. In rats challenged to induce arthritis, we started treatment with a β_2_-AR agonist treatment at disease onset and continued daily treatments until severe disease. In severe disease, we harvested lymph nodes that drain (DLNs) the arthritic joints and the spleen, organs where arthritogenic T cells develop. Additionally, mesenteric lymph nodes (MLNs) were also harvested—sites where T lymphocytes that do not transfer the disease to naïve animals reside. In vivo treatment with a β_2_-AR agonist (terbutaline) altered IFN-γ production by T cells differently in each lymphoid organ examined. Ex vivo assessments of T cell cytokine production revealed that β_2_-AR stimulation decreased IFN-γ in MLNs, induced no change in IFN-γ in splenocytes, and increased IFN-γ production in DLN cells from arthritic rats compared to untreated arthritic rats [32,124]. These findings are consistent with β_2_-ARs signaling via cAMP in MLNs, ERK1/2 in DLNs and receptor desensitization/down-regulation in the spleen. Receptor down-regulation and desensitization were confirmed in the spleen using receptor binding assays and assessing β_2_-AR induced effects on cAMP production in arthritic and non-arthritic rats [32].

Support for a shift in receptor signaling from PKA to ERK1/2 was demonstrated in DLN cells from arthritic rats by assessing the phosphorylation of β_2_-ARs by PKA and GRK [32]. The phosphorylation of β_2_-ARs by PKA increases during severe disease and then later declines during chronic disease. In contrast, the β_2_-AR phosphorylation by GRKs increases during severe and chronic disease [32]. This observation coupled with an increase in IFN-γ production is consistent with the phosphorylation of β_2_-AR by GRK5/6, recruitment of β-arrestin 2 and signaling of the receptor via ERK1/2. In contrast to our findings in DLN cells, elevated pβ_2_-AR by GRK in splenocytes was restricted to the chronic disease phase [32]. This coupled with the inability of β_2_-AR agonists to increase cAMP or to alter IFN-γ production is consistent with pβ_2_-AR by PKA and GRK2-induced receptor desensitization, and β-arrestin 2-mediated receptor down-regulation. Future studies are required to confirm GRK5/6-induced β-arrestin 2-mediated signaling via ERK in DLN cells and GRK2-induced β-arrestin 1-mediated receptor desensitization and down-regulation. We will also need to determine whether β_2_-agonist-induced increases in IFN-γ in DLN cells can be blocked by ERK1/2 inhibitors. 

The different β_2_-AR functions observed in the DLN and spleen in arthritic rats is intriguing. In future studies, we will pursue the possibility that the local environment is important for the different signaling pathways in these two immune organs given that the DLN receive lymphatic drainage from the inflamed joints, whereas the spleen filters the blood. In addition, a similar pattern of β_2_-AR signaling was observed in rats challenged with the bacterial cell wall component of the adjuvant used to induce arthritis, indicating a role for Toll-like receptors. Finally, preliminary data from our laboratory indicate that differentially located central premotor neurons in the rostroventral medulla regulate sympathetic firing rates in the DLN and spleen, which may contribute to disease-mediated differences in β_2_-AR signaling in these two secondary lymphoid organs [194]. 

## 7. β-AR Promiscuity in the Autoimmune IMID, RA: A Consequence of High SNS Tone in the SNS-Splenic Axis?

An important function of the SNS is to respond to danger signals, like injury-protective heat shock proteins or in response to noninfectious foreign substances or injury. The SNS serves as an integrator of these stimuli and subsequent regulator of inflammatory machinery in a normal resolving immune response, as well as sterile inflammation—low-grade chronic linked with aging, and chronic systemic inflammatory disease regardless of etiology. Diverse IMIDs, such as autoimmune diseases, chronic heart failure and COPD evoke a systemic inflammatory response mediated by an anti-inflammatory sympathetic-splenic-pathway.

A repeated finding across most chronic inflammatory diseases where the SNS has been studied is dysregulation of the SNS, most commonly nerve hyperactivity [45,46,47,48,49,50,194]. The response of SNS to systemic inflammation is context-dependent, and determined by the integration of signals activated by the inflammagen(s), and is mediated via splanchnic nerves that target postganglionic neurons in the celiac and superior mesenteric ganglion that innervate and regulate cells of the immune system that reside in the spleen. Cross-regulation of chronic inflammation and the SNS can induce hyperactivity of sympathetic nerves in the spleen. Chronically high SNA and systemic inflammation can drive comorbid organ damage [195,196,197,198,199,200,201,202,203,204] as well as SNS pathology. Our laboratory has been unraveling the basic mechanisms by which high SNA dysregulates immune function to precipitate the onset of disease and systemic inflammation using a chronic inflammatory disease model with autoimmune mechanisms as a model for IMIDs (see Figure 2). Using autoimmune-mediated arthritis as a primary model system, our data support that hyper-SNA in the spleen and relevant lymph nodes can drive non-pathogen chronic inflammatory processes by changing β_2_-AR signaling to a non-canonical pathway, in response to non-infectious activation.

We and others using RA patients or animal models of inflammatory arthritis verify that SNS-immune communication is significantly altered in several functionally significant ways [32,112,113,114,115,116,117,118,119,120,121,122,123,152,205]. In rodent models of IA, both the spleen and lymph nodes that drain the hind arthritic limbs (DLNs) display sympathetic nerve pathology [113,206] and altered β_2_-AR functions [32,124]. Thus, a vital efferent pathway is compromised in IA. Similarly, dysautonomia involving efferent sympathetic pathways occurs in IMIDs listed in Table 1 [207,208,209,210,211,212,213,214,215,216,217,218].

That stressors that increase SNA can trigger and worsen clinical disease is supported by a strong relationship between onset of autoimmunity and severe stress in 80% of patients, and dual autonomic and immune dysregulation [42,44,219,220,221,222,223]. SNS tone is high in IA patients [224,225,226]. Thus, studies are warranted to test this hypothesis. We have shown that presympathetic RVLM neurons that express I_1_Rs control splenic SNA, and I1R agonists lower splenic SNA [227,228]. Although proposed in IA and other IMIDs, high SNA as a cause for disease has not been directly tested. However, we have reported nerve pathology and β_2_-AR desensitization in IA, findings consistent with chronic high SNS tone in IMIDs [32,111]. A Nobel Prize awarded discovery [229] proved that β_2_-AR and other G-coupled protein receptors can shift their signaling from cAMP to the extracellular signal-regulated kinases (ERK)1/2 pathway in cell lines and cardiomyocytes [229,230] by a switch inducible by high NE concentrations [164,231,232]. Our findings indicate that sympathetic splenic nerves remodel and spleen cells lose β_2_-AR signal transduction via cAMP (Figure 4A). In DLN cells, β_2_-AR signaling via ERK1/2 occurs due to altered receptor phosphorylation and aberrantly increases IFN-γ production to drive disease [32]. Our data indicate that the bacterial cell wall component of complete Freund’s adjuvant mediates this effect. The changes in splenocyte and DLN β_2_-AR signaling remove the normal inhibitory influence of the SNS on inflammation and Th1 cells, skewing balance between suppressive Treg cells and differentiating Th1 cell that drives disease [233,234]. Therefore, our collective data support that dysautonomia resulting in high sympathetic tone induces an imbalance in differentiating Treg cells required for tolerance and Theff cells that promotes autoimmunity.

Our early work revealed altered DLN NE concentrations, but not spleen of IA rats despite nerve retraction/remodeling [111,113]. Findings in spleen suggest higher SNA to compensate for lost neural input. Nerve injury, higher NE concentration (DLNs) and abnormal β_2_-AR functions (DLN and spleen) are indicative of high SNA and dysfunction. Later, we were the first to show that the opposing effects of α- and β-AR drug treatments relative to disease onset [118,120,235] are due to different immune response phases (initiation vs effector) [112], a finding verified in the CIA model of IA [122]. The studies are consistent with high splenic SNA in IA and dysautonomia in an IMID. Similar defects are reported in the cardiovascular system and other adrenergic targets as a result of chronic β_2_-AR stimulation from high SNA [236,237,238,239]. Collectively, research supports SNS involvement in disease induction and progression in IMIDs. Moreover, they suggest treatment of IMIDs via adrenergic ligands may not be feasible in patients due to opposing effects during distinct phases of disease.

## 8. Concluding Remarks and Potential Significance

Research supports severe life stressors commonly precede the onset of many IMIDs, including those with autoimmune-mediated mechanisms, with reports of up to 80% of patients reporting a severe life stressor before disease onset [42,43,44]. Dysautonomia, particularly high SNA, is linked to many age-related autoimmune and metabolic IMIDs (e.g., [6,7,11,18,22,23,24,42,43,44,45,46,47,48,49,50,51,52,53,54]). Despite this strong linkage, it remains unknown how stress and high SNA trigger disease onset and chronic inflammation. Findings presented in a prototypical IMID model lend support to our hypothesis that chronic high splenic SNA before disease onset precipitates SNS pathology and can sabotage the normal resolution of cellular immunity and subsequent return to an anti-inflammatory state. A significant consequence of hyper-SNA and chronic inflammation in IMIDs is their propensity to secondarily induce comorbid organ damage [195,196,197,198].

It is formidable to understand IMID pathology due to individual complexities of the SNS-immune system, their “cross-talk” and secondary involvement of multiple organ systems. However, overcoming this challenge may afford a great opportunity for rational neurally-based prevention strategies not yet seriously considered. The advantages of this treatment approach are many, including reduced comorbidities that result from overactivation of stress pathways, and lower costs to treat with fewer adverse side effects compared with the currently used non-selective immunosuppressive biologic therapeutics. To this end, our research has begun to unravel basic mechanisms by which high SNA dysregulates immune functions that can precipitate the onset of disease and subsequent chronic inflammation in a prototypical IMID model. Our findings reveal important differences in sympathetic regulation of immune response in spleen versus the LNs that drain disease-targeted tissues. These findings are consistent with the divergent functional roles these secondary immune organs carry out.

**Figure 5:**
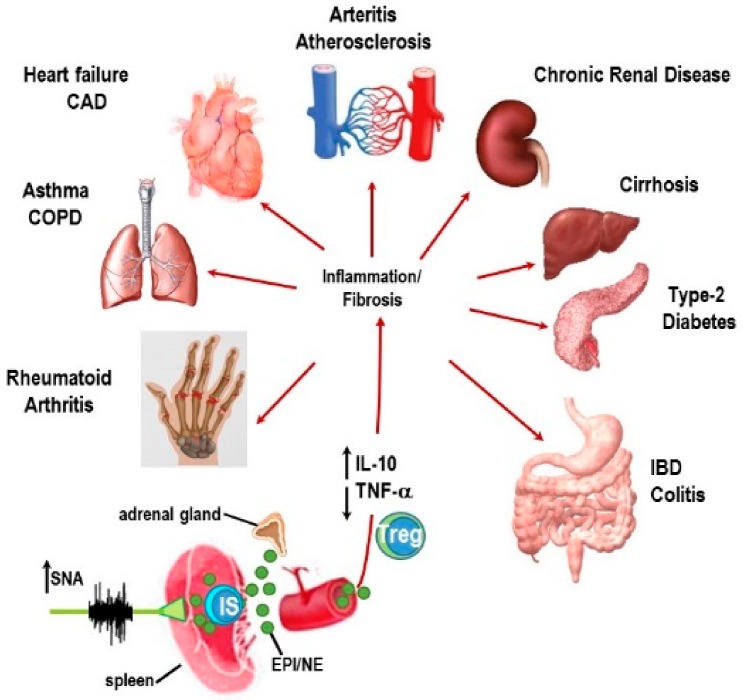
One mechanism is proposed whereby high sympathetic nerve activity (SNA) in the spleen may dampen systemic inflammation (red arrows) in immune-mediated inflammatory diseases (IMIDs). In the spleen, a β_2_-AR-mediated increase of interleukin-10 (IL-10, upward arrow) secretion and inhibition of tumor necrosis factor (TNF-α, downward arrow) by splenic immunocytes into the systemic circulation exert anti-inflammatory actions in tissues and organs (indicated by red arrows) that are targeted by IMIDs, including inflammatory bowel diseases (IBD) such as colitis, type-2 diabetes, cirrhosis, chronic renal disease, arteritis, atherosclerosis, chronic heart failure, coronary artery disease (CAD), asthma, chronic obstructive pulmonary disease (COPD), and rheumatoid arthritis.

Moreover, our research reveals divergence from the neurotypical β_2_-AR-PKA-mediated signaling profile that is secondary immune organ-dependent. In the spleen, our findings reveal a normal down-regulatory response of β_2_-ARs that is consistent with our previous reports of high SNA and sympathetic neuropathy in the spleens of arthritis rats. An important function of the spleen is to detect foreign antigens in the bloodstream that escape detection/elimination by local phagocytes and regionally located lymph nodes. Assisting this splenic function, the ANS is part of the “supporting cast”. The SNS plays a supporting role, not only in antigen elimination, but also in alerting and modifying other organ system functions, meeting organ-specific changes in energy demands, and in providing protection against inflammation-induced bystander tissue damage in organ systems that are critical for survival [240]. Splenic macrophages and other cell populations (i.e., regulatory B cells) secrete the anti-inflammatory cytokine IL-10 into the circulation, suppressing pro-inflammatory cytokine-induced organ damage [78,241]. The spleen is a main player in preventing visceral organ dysfunction in IMIDs (Figure 5) [242]. A primary role for the spleen is also emerging outside of the prevalent IMIDs, as metabolic diseases are now considered as IMIDs (e.g., obesity and metabolic syndrome [240,241,242,243,244]). These metabolic IMIDs are strongly associated with secondary organ damage/dysfunction in kidney, liver, heart, adipose tissue, lungs, and blood vessels that increase morbidity and mortality, and significantly contribute to poorer quality of life and high healthcare burden worldwide.

In contrast to the spleen, in draining LNs, β_2_-AR signaling switches from its coupling with PKA signal transduction to its coupling with the ERK1/2 pathway, a major pathway for mediating chronic local inflammation. As mentioned above, the transfer of either spleen or DLN cells into a naïve animal induce disease. However, rodents immunized with spleen cells from arthritic syngeneic animals are more effective in transferring disease. However, the DLNs are important for disease development, because removal of the DLNs within seven days of an arthritis-inducing challenge prevents disease development. Whether removing the spleen within this period would also prevent disease has not been determined. This surprising response may result from a functional priority of the spleen for survival in the face of chronic inflammation. These findings underscore the importance of the spleen in disease pathology, and as a target for disease prevention or intervention, and support continued investigation of the role of the spleen in IMIDs. Similar findings that β_2_-AR can promiscuously shift signal transduction to intracellular pathways that more directly regulate immune functions have been reported by Lefkowitz and coworkers [141,164] in chronic heart failure, a non-autoimmune model of an IMID. 

Harmon et al. [245] have reported findings suggestive that PKA-independent mechanisms may play a role in chronic inflammatory diseases of the bladder, such as interstitial cystitis [245]. Collectively, our findings and others lend support for a role of the SNS in regulating IMIDs through its regulation of common inflammatory pathways (i.e., MAPK and ERK). Finally, our data suggest that a single therapeutic target would likely need to target the central nervous system [120,124,246]. Available data indicate that monodrug therapy that targets β_2_-ARs after disease onset significantly lessens disease outcome, but does not prevent disease, likely because of the divergence in β_2_-AR signaling in the spleen and lymph nodes, which produces anti- and pro-inflammatory cytokine profiles, respectively. Importantly, these adrenergic drug therapies were as effective as Enbrel in treating inflammatory arthritis in our rodent model. If the same effects are realized in patients with RA, then normalizing SNS tone would have better safety profiles, be easier to administer, and more cost effect than the currently used biologics.

The SNS is an often overlooked target in the treatment of IMIDs, but the ANS has the potential to significantly enhance basic knowledge about neuropathology that impairs SNS-immune cross-regulation in IMIDs, and provide insight into approaches for better monitoring, prevention and/or treatment. Ultimately our research could lower morbidity and mortality, improve health care and quality of life, better maintain a healthy workforce, and reduce medical costs. This research could have significant rewards as over 130 million Americans suffer from one or more IMIDs, and it is anticipated that 81 million people will suffer from multiple IMIDs by 2020 [247,248]. Higher incidence is forecasted for virtually every IMID. Importantly, we could hasten the path to clinical use of SNA-reducing drugs. Many FDA-approved sympatholytic drugs are available off-label to treat IMIDs, providing a rationale for clinical trials. Alternatively, neurostimulation of neural sites that exert sympatholytic activity is becoming a promising alternative to biologics and other adrenergic drug treatments. One prominent pathway with potential anti-inflammatory effects is the vagal suppression of the SNS. 

Our studies have focused on dysautonomia and its impact on systemic inflammation and skewed Th cell balance, and the spleen’s contribution to it. Our published data provide compelling support for IA as an IMID model that can be exploited to better understand how dysautonomia and persistent inflammation induce disease and increase autoimmunity risk. Dysautonomia plays a significant role in the pathophysiology of diseases hallmarked by chronic inflammation. The fact that cytokine-targeted therapy, such as Enbrel, effectively treats multiple inflammatory conditions of unknown origin confirms this paradigm. Immune-targeted therapies, such as Enbrel, have significantly improved health care for IMID patients, mostly from reduced inflammation and pain. However, many patients are/become refractory, and all have greater risk for cancer and fatal opportunistic infections [249,250,251]. Our preclinical research provides robust evidence that neurally-based approaches can have translational value similar to Enbrel by normalizing or restoring autonomic-immune homeostasis, but without the adverse side effects of cytokine-targeted therapies.

## Figures and Tables

**Table 1:** Examples of IMIDs, their Etiology and Neural-Immune Features

Examples of IMIDs *	Etiology	Chronic Inflammation		Th Cell Immune	Dysregulation of Stress Pathways	
		Main Target	Systemic	Activation Profile	ANS	HPA
Ankylosing spondylitis	Autoimmune?	Joints of spine	yes	Th2/Th17	yes	yes↓
Arteritis/Vasculitis	Autoimmune?	Blood vessels	yes	Th17/Treg	yes	yes↓
Behcet’s disease	Autoimmune?	Blood vessels	yes	Th1/Th17	yes	yes↓
**Inflammatory bowel disease**						
**Crohn’s disease**	Unknown	Gastrointestinal tract	yes	Th1/Th17	yes	yes↓
**Ulcerative colitis**	Autoimmune?	Colon and rectum	yes	Th1/Th17	yes	
Juvenile idiopathic arthritis	Autoimmune?	Joints	yes	Th1/Th17	yes	yes↓
Multiple Sclerosis	Autoimmune?	CNS myelin	yes	Th17/Treg	yes	
**Psoriasis**	Autoimmune?	Skin	yes	Th1/Th2	yes	yes↓
**Psoriatic arthritis**	Autoimmune?	Skin and joints	yes	Th1/Th17	yes	yes↓
**Rheumatoid arthritis**	Autoimmune?	Joints	yes	Th1/Th17	yes	yes↓
Sarcoidosis	Autoimmune?	Lungs, skin	yes	Th1/Th17	yes	yes↓
**Systemic lupus erythematosus**	Autoimmune?	Skin, joints, visceral organs	yes	Th1/Th17	yes	yes↓
Type 1 diabetes mellitus	Autoimmune?	β-cells in pancreas	yes	Th1/Th17	yes	–
Uveitis	Autoimmune?	Uvea (iris, ciliary body & choroid)	yes	Th17		

* The most common occurring IMIDs are bolded. ↓, hypofunction. ANS, autonomic nervous system, HPA, hypothalamic–pituitary–adrenal, Th, T helper cell.
